# Genetic variants in RhoA and ROCK1 genes are associated with the development, progression and prognosis of prostate cancer

**DOI:** 10.18632/oncotarget.15197

**Published:** 2017-02-08

**Authors:** Kang Liu, Xiao Li, Jie Wang, Yichun Wang, Huiyu Dong, Jie Li

**Affiliations:** ^1^ Department of Urology, The First Affiliated Hospital of Nanjing Medical University, Nanjing, China; ^2^ Department of Urology, The Affiliated Cancer Hospital of Jiangsu Province of Nanjing Medical University, Nanjing, China

**Keywords:** genetic variants, RhoA/ROCK1 pathway, prostate cancer, prognosis, biochemical recurrence

## Abstract

The contribution of genetic variants in RhoA and ROCK1 genes towards prostate cancer risk has not been reported before. We genotyped six potentially functional genetic variants in a case-control study of 1699 subjects. Overall, we found rs2410 mutant allele and rs2269736 wild allele were risk factors for prostate cancer. Individuals carrying more than two risk alleles were exposed to hazard of prostate cancer. In addition, we demonstrated that the risk of biochemical recurrence might be linked with clinico-pathological characteristics and also genetic factors. Unfortunately, no associations were observed between all polymorphisms and clinico-pathological characteristics. Moreover, no genotype was found as significant independent prognostic predictor for biochemical recurrence survival in Multivariate Cox regression analysis after Bonferroni correction. Our study is the first to clarify the relations of genetic variants of RhoA and ROCK1 genes with development, progression and prognosis of prostate cancer. These variants may be promising novel biomarkers to facilitate clinical treatment decision-making.

## INTRODUCTION

Prostate cancer (PCa) is a multifactorial and complex disease [1]. In the United States, there was estimated to be 180,890 new cases in 2016 [1]. In contrast, a lower incidence rate was observed in Asian region than in western countries [2]. However, the occurrence of PCa has continuously increased in China recently, mainly owing to advocacy of massive diagnostic screening, active transrectal prostate biopsies and considerable media attention [3]. Meanwhile, prostate cancer is reported to have unique characteristics, such as hormonal sensitivity, racial difference in prevalence and hereditary susceptibility [4]. These clinical discrepancies may reflect underlying heterogeneity in disease etiology and have influence on screening, treatment, and prognosis. Unfortunately, the etiology and pathogenesis remain a puzzle of PCa at present [5]. In addition, another clinical challenge is to distinguish indolent and aggressive PCa. Different prognoses are extremely important to facilitate clinical treatment decision-making. As we all known, radical prostatectomy (RP) is a recommended option with curative purpose for localized PCa. However, more than 30% of men undergoing RP will suffer from disease relapse [6, 7]. Hence, we need to identify novel prognostic markers to better predict the incidence and progression of PCa and the risk of biochemical recurrence (BCR) in order to guide comprehensive individual therapy.

Small guanosine triphosphate hydrolases (GTPase) of the Ras homology (Rho) family work as molecular switches in a wide variety of signal transduction pathways that regulate diverse cellular functions [8]. RhoA, a member of Rho GTPases superfamily, is a GTP/GDP-binding protein which cycles from a GDP-bound inactive state to a GTP-bound active state [9]. Malignant tumor cells display uncontrolled cell cycle and proliferation, enhanced migratory properties as well as escape of programmed cell death [10]. Published papers have confirmed that RhoA could stimulate the formation of stress fibers and focal adhesions and negatively regulate the levels of the cell cycle inhibitors p21cip1 and p27kip1 [11, 12]. Also, activation of RhoA leads to Fas-dependent apoptosis of Jurkat cells and elevated cell survival during zebrafish embryogenesis [13, 14]. Rho associated, coiled-coil containing protein kinase 1 (ROCK1), a key downstream effector of the small GTPase RhoA, is ubiquitously expressed throughout embryogenesis and in most tissues [15]. It is deemed to be involved in varied cellular processes including actin cytoskeletal organization, cell-cell adhesion, migration, invasion, transformation, mitosis and apoptosis [16–18].

Many lines of evidence have proved that RhoA/ROCK1 signaling pathway participates in PCa pathogenesis. Somlyo AV and colleagues stated that invasiveness of human PCa was facilitated by the Rho/ROCK1 pathway and inhibition of RhoA or its interacting protein ROCK1 might diminish motility of PCa cells [19]. Similar conclusions were drawn by Sequeira L as well [20]. Besides, Rho/ROCK1 pathway was found to regulate membrane androgen receptor-induced apoptosis in both LNCaP and DU145-prostate cancer cells [21]. Therefore, it is rational to speculate that RhoA/ROCK1 signaling pathway is likely to be an ideal candidate target in PCa prediction and treatment.

However, the contribution of genetic variants in RhoA and ROCK1 genes towards the risk of PCa has not been reported. Given the significant role of RhoA and ROCK1 in the development of PCa, we conceived genetic variants in RhoA/ROCK1 signaling pathway as appropriate biomarkers of PCa. To verify this hypothesis, we selected six potentially functional single nucleotide polymorphisms (SNPs) in RhoA and ROCK1 genes to detect whether these genetic variants could forecast progression and recurrence of PCa.

## RESULTS

### Characteristics of the study population

The demographic characteristics and the clinical information of 830 PCa patients and 869 controls were summarized in Table 1. In short, there were no statistically significant differences between cases and controls with regards to age, BMI, drinking status, hypertension and diabetes. However, there were more smokers and tea drinkers among the cases than among the controls (*P* = 0.002 and *P* = 0.040, respectively). Moreover, in comparison with the controls, a significantly higher proportion of the PCa patients had family history of cancer (*P* < 0.001). Among cases, 498 (60.0%) patients were in the localized stage and 332 (40.0%) patients were in the advanced stage. The percent of subjects in Gleason score < 7, = 7 and > 7 subgroups was 28.1%, 36.4% and 35.5%, respectively. Furthermore, 58.2% of 830 patients had a PSA level greater than 20 ng/ml, whereas, 20.7% and 21.1% patients had a level between 10 ng/ml to 20 ng/ml and less than 20 ng/ml, respectively.

**Table 1:** Frequency distributions of selected variables between the PCa cases and controls

Variables	cases (*n* = 830)		controls (*n* = 869)		*P*^a^
	*n*	%	*n*	%	
**Age (years) (Mean ± SD)**	71.2 ± 7.9		71.0 ± 6.3		0.695
**≤ 71**	383	46.1	429	49.3	0.184
**> 71**	447	53.9	440	50.7	
**BMI (kg/m2) (Mean ± SD)**	23.7 ± 7.8		23.4 ± 2.9		0.906
**Smoking status**					
**Never**	325	39.2	392	45.1	**0.002^b^**
**Ever**	505	60.8	447	54.9	
**Pack-years of smoking**					
**0**	325	39.2	392	45.1	**0.027^b^**
**0–20**	229	27.6	232	26.7	
**> 20**	276	32.4	245	28.2	
**Drinking status**					
**Never**	582	70.1	638	73.4	0.131
**Ever**	248	29.9	231	26.6	
**Hypertension**					
**No**	614	74.0	667	76.8	0.184
**Yes**	216	26.0	202	23.2	
**Diabetes**					
**No**	707	85.2	761	87.6	0.151
**Yes**	123	14.8	108	12.4	
**Tea drinking**					
**Never**	407	49.0	383	44.1	**0.040^b^**
**Ever**	423	51.0	486	55.9	
**Family history of cancer**					
**No**	639	77.0	747	86.0	**< 0.001^b^**
**Yes**	191	23.0	122	14.0	
**Clinical stage**					
**Localized**	498	60.0	–	–	
**Advanced**	332	40.0	–	–	
**Gleason score**					
**< 7**	233	28.1	–	–	
**= 7**	302	36.4	–	–	
**> 7**	295	35.5	–	–	
**PSA (ng/ml)**					
**≤ 10**	175	21.1	–	–	
**10–20**	172	20.7	–	–	
**> 20**	483	58.2	–	–	

^a^Two-sided *χ*^2^ test for the frequency distributions of selected variable between cases and controls.

^b^Bold values indicated significant differences between two groups.

### Genotype and allele frequencies of RhoA/ROCK1 polymorphisms in PCa cases and controls

Genotype distributions and allele frequencies of these polymorphisms in cases and controls were listed in Table 2. The observed genotype frequencies of all SNPs among the controls confirmed to the HWE (*P* > 0.05). Overall, the single loci analysis only demonstrated that the genotype frequency of RhoA rs2269736 was crucially different between the cases and controls (*P* < 0.001). Moreover, for RhoA rs2269736, multivariate logistic regression analysis also revealed that the genotype and allele distributions were remarkably different between cases and controls in the heterozygote model (adjusted OR = 0.63, 95% CI = 0.48–0.82), the homozygote model (adjusted OR = 0.76, 95% CI = 0.69–0.83), the dominant model (adjusted OR = 0.79, 95% CI = 0.72–0.86) and the allele comparison (adjusted OR = 0.66, 95% CI = 0.57–0.76). Additionally, multivariate logistic regression analysis indicated that individuals carrying RhoA rs2410 CC genotype had a significantly higher incidence of PCa than those carrying AA genotype. However, such conclusion in RhoA rs2410 was not established with Bonferroni correction. Unfortunately, *P* value was adjusted as 0.05/3 with Bonferroni correction, and no positive relationships with PCa risk were detected in RhoA rs2410, RhoA rs2625955, ROCK1 rs11874761, ROCK1 rs35996865 and ROCK1 rs8089974.

**Table 2:** Genetic variants in the RhoA/ROCK1 pathway associated with the PCa risk

Genetic Variants	Case (*n* = 830)		Control (*n* = 869)		*P*^a^	Adjusted OR (95% CI)^b^
	*n*	%	*n*	%		
RhoA rs2410						
AA	199	24.0	233	26.8	0.059	1.00 (reference)
AC	378	45.5	415	47.8	0.591	1.06 (0.84–1.35)
CC	253	30.5	221	25.4	**0.028**	**1.11 (1.01–1.21)**
AC+CC	631	76.0	636	73.2	0.180	**1.11 (1.02–1.21)**
A allele	776	46.8	881	50.7	**0.022**	1.00 (reference)
C allele	884	53.2	857	49.3		**1.18 (1.03–1.35)**
RhoA rs2625955						
AA	325	39.2	348	40.1	0.849	1.00 (reference)
AC	376	45.3	394	45.3	0.938	0.99 (0.80–1.22)
CC	129	15.5	127	14.6	0.567	1.01 (0.92–1.12)
AC+CC	505	60.8	521	59.9	0.708	1.01 (0.92–1.12)
A allele	1026	61.8	1090	62.7	0.585	1.00 (reference)
C allele	634	38.2	648	37.2		1.01 (0.88–1.17)
RhoA rs2269736						
AA	186	22.4	116	13.3	**< 0.001**	1.00 (reference)
AG	399	48.1	404	46.5	**< 0.001**	**0.63 (0.48–0.82)**
GG	245	29.5	349	40.2	**< 0.001**	**0.76 (0.69–0.83)**
AG+GG	644	77.6	753	86.7	**< 0.001**	**0.79 (0.72–0.86)**
A allele	771	46.5	636	36.6	**< 0.001**	1.00 (reference)
G allele	889	53.5	1102	63.4		**0.66 (0.57–0.76)**
ROCK1 rs11874761						
AA	12	1.4	11	1.3	0.914	1.00 (reference)
AG	169	20.4	182	20.9	0.708	0.80 (0.34–1.90)
GG	649	78.2	676	77.8	0.761	0.93 (0.71–1.23)
AG+GG	818	98.6	858	98.7	0.748	0.99 (0.88–1.11)
A allele	193	11.6	204	11.7	0.920	1.00 (reference)
G allele	1467	88.4	1534	88.3		0.97 (0.79–1.20)
ROCK1 rs35996865						
GG	16	1.9	15	1.8	0.140	1.00 (reference)
GT	179	21.6	155	17.8	0.833	1.03 (0.48–2.18)
TT	635	76.5	699	80.4	0.658	0.94 (0.74–1.20)
GT+TT	814	98.1	854	98.2	0.756	**0.89 (0.80–0.99)**
G allele	211	12.7	185	10.6	0.061	1.00 (reference)
T allele	1449	87.3	1553	89.4		**0.80 (0.65–0.99)**
ROCK1 rs8089974						
GG	8	1.0	14	1.6	0.119	1.00 (reference)
GT	174	20.9	153	17.6	0.126	2.01 (0.80–5.08)
TT	648	78.1	702	80.8	0.278	1.16 (0.86–1.56)
GT+TT	822	99.0	855	98.4	0.238	0.93 (0.83–1.04)
G allele	190	11.5	181	10.4	0.335	1.00 (reference)
T allele	1470	88.5	1557	89.6		0.89 (0.71–1.10)

Bold values indicated significant differences between two groups.

^a^Two-sided *χ*^2^ test for either genotype distributions or allele frequencies between the cases and controls. All *P* values were Bonferroni corrected, and statistical significance was set at *P* < 0.01667 (0.05/3).

^b^Adjusted for age, bmi, pack-years of smoking, drinking status, tea drinking, hypertension and diabetes in logistic regression model.

### Combined analysis of RhoA rs2410 and rs2269736

Considering latent combined effects from different variants or genotypes and potential interactions of RhoA gene polymorphism on the risk of PCa, we combined these two tSNPs based on the numbers of risk alleles (that is, rs2410 C and rs2269736 A alleles). As shown in Table 3A, when compared with individuals carrying 0 risk allele, those who carrying 1 or 2 risk alleles had no obvious PCa susceptibility. Yet, subjects with 3 or 4 risk alleles were exposed to hazard of PCa. Given that the relatively small sample size of reference group (95 cases and 125 controls) were likely to weaken the statistic power, we subsequently dichotomized the combined risk alleles into two groups. Results showed that subjects carrying two to four risk alleles had significantly increased risk of PCa, relative to those with 0 or 1 risk alleles (adjusted OR =1.37, 95% CI = 1.12–1.68).

**Table 3A:** Frequency distributions of the number of risk alleles between cases and controls, and their association with PCa risk

	cases (*n* = 830)		controls (*n*= 869)		*P*^a^	Adjusted OR (95% CI)^b^
	*n*	%	*n*	%		
Number of risk alleles						
**0**	95	11.5	125	14.4	**< 0.001**	1.00 (reference)
**1**	175	21.1	223	25.7	0.850	1.09 (0.77–1.53)
**2**	314	37.8	354	40.7	0.324	1.10 (0.94–1.28)
**3**	132	15.9	106	12.2	**0.009**	**1.19 (1.05–1.35)**
**4**	114	13.7	61	7.0	**< 0.001**	**1.29 (1.16–1.43)**
**Recombined groups**						
**0-1**	270	32.5	348	40.0	**0.001**	1.00 (reference)
**2-4**	560	67.5	521	60.0		**1.37 (1.12–1.68)**

**Table 3B:** Frequency distributions of the combined genotypes of rs2410 and rs2269736 among all subjects, and their association with PCa risk

**Combined genotype**						
**AAAA**	39	4.7	31	3.5		1.00 (reference)
**AAAG**	65	7.8	77	8.9	0.173	0.71 (0.38–1.35)
**AAGG**	95	11.5	125	14.4	0.067	0.76 (0.57–1.00)
**ACAA**	33	4.0	24	2.8	0.805	1.05 (0.81–1.37)
**ACAG**	235	28.3	245	28.2	0.291	0.93 (0.82–1.06)
**ACGG**	110	13.3	146	16.8	0.058	0.91 (0.81–1.01)
**CCAA**	114	13.7	61	7.0	0.169	1.08 (0.98–1.20)
**CCAG**	99	11.9	82	9.4	0.884	1.00 (0.92–1.09)
**CCGG**	40	4.8	78	9.0	**0.003**	**0.88 (0.81–0.96)**

Bold values indicated significant differences between two groups.

^a^Two-sided *χ*^2^ test for either genotype distributions or allele frequencies between the cases and controls. All *P* values were Bonferroni corrected, and statistical significance was set at *P* < 0.00556 (0.05/9).

^b^Adjusted for age, bmi, pack-years of smoking, drinking status, tea drinking, hypertension and diabetes in logistic regression model.

We then conducted combined genotype analysis to further probe underlying combined effects in the development of PCa. The outcomes of combined genotype analysis were outlined in Table 3B. When using AAAA combined genotype as reference, we found CCGG was the most protective genotype (adjusted OR = 0.88, 95% CI = 0.81–0.96) while AAGG showed a boundary protective effect (adjusted OR = 0.76, 95% CI = 0.57–1.00). However, to some extent, no other positive results were obtained on account of limitation of insufficient sample size. Though *P* value was adjusted as 0.05/9 with Bonferroni correction, such conclusions were also tenable.

### Stratification analysis of the association between RhoA/ROCK1 polymorphisms and PCa

Although not all genetic variants in RhoA and ROCK1 genes were proved to be related to overall risk of PCa, we further conducted subgroup analysis stratified by age, smoke status, Pack-years of smoking, drink status, tea drinking, family history of cancer, hypertension and diabetes. All results of stratification analysis were summarized in Supplementary Table 1A–1F. Briefly, the increased PCa risk connected with rs2410 was more prominent among older healthy subjects without habit of alcohol and tea. The association between rs2269736 and a decreased PCa risk was relatively weaker among non-smokers. Interestingly, we also discovered that ROCK1 rs8089974 and rs35996865 were protective factors in the midst of diabetics. However, after Bonferroni correction with *P* value adjusted as 0.05/3, such conclusions were invalid in some subgroups of particular genes.

Similarly, we performed stratified analysis to survey the relationship between the numbers of risk alleles and PCa susceptibility (Table 4). As a result, the correlation between combined risk alleles and PCa risk was more evident in healthy older individuals (adjusted OR = 1.42, 95% CI = 1.08–1.88) who were heavy smoker (adjusted OR = 1.89, 95% CI = 1.29–2.77), non-drinker (adjusted OR = 1.36, 95% CI = 1.07–1.73), and without family history of cancer (adjusted OR = 1.39, 95% CI = 1.11–1.74).

**Table 4:** Stratification analysis of number of risk alleles and PCa risk

Variables	Case (*n* = 830)		Control (*n* = 869)		*P*^a^	Adjusted OR (95% CI)^b^
	Number of risk alleles		Number of risk alleles			
	0–1	2–4	0–1	2–4		
**Total**	270	560	348	521	**0.001**	**1.37 (1.12–1.68)**
**Age**						
**≤ 71**	127	256	169	260	0.706	1.32 (0.98–1.78)
**> 71**	143	304	179	261	**0.007**	**1.42 (1.08–1.88)**
**Smoke status**						
**Never**	112	213	157	235	0.124	1.25 (0.91–1.71)
**Ever**	158	347	191	286	**0.004**	**1.47 (1.12–1.94)**
**Pack-years of smoking**						
**0–20**	80	149	85	147	0.703	1.15 (0.77–1.72)
**> 20**	78	198	106	139	**< 0.001**	**1.89 (1.29–2.77)**
**Drinking status**						
**Never**	191	391	256	382	**0.008**	**1.36 (1.07–1.73)**
**Ever**	79	169	92	139	0.069	1.31 (0.89–1.93)
**Tea drinking**						
**Never**	122	285	151	232	**0.005**	**1.56 (1.15–2.10)**
**Ever**	148	275	197	289	0.086	1.21 (0.92–1.59)
**Family history of cancer**						
**No**	205	434	300	447	**0.002**	**1.39 (1.11–1.74)**
**Yes**	65	126	48	74	0.340	1.28 (0.79–2.09)
**Hypertension**						
**No**	204	410	268	399	**0.013**	**1.34 (1.06–1.69)**
**Yes**	66	150	80	122	0.074	1.46 (0.96–2.20)
**Diabetes**						
**No**	230	477	300	461	**0.006**	**1.32 (1.06–1.64)**
**Yes**	40	83	48	60	0.063	1.72 (0.97–3.04)

Bold values indicated significant differences between two groups.

^a^Two-sided *χ*^2^ test for either genotype distributions or allele frequencies between the cases and controls. All *P* values were Bonferroni corrected, and statistical significance was set at *P* < 0.00556 (0.05/9).

^b^Adjusted for age, bmi, pack-years of smoking, drinking status, tea drinking, hypertension and diabetes in logistic regression model.

We further conducted stratified analysis among PCa patients according to some disease-related clinical features including clinical stage, Gleason score and PSA level. Regrettably, no statistical evidence was explored for any interactions between single SNPs or combined genotypes with PCa risk (Supplementary Table 2A–2G).

### Effects of RhoA/ROCK1 polymorphisms on PCa biochemical recurrence

In order to seek possible effects of RhoA/ROCK1 polymorphisms on PCa prognosis, we conducted analysis of BCR in a cohort of 289 patients who accepted radical prostatectomy. All clinico-pathological characteristics of study populations were shown in Supplementary Table 3. In brief, 134 patients were diagnosed with PCa with a < 10 ng/ml PSA level. Nearly 70% presented with a Gleason score ≤ 7. About 20% of patients had nodal invasion and positive margin. All patients included did not accept hormonotherapy.

**Figure 1:**
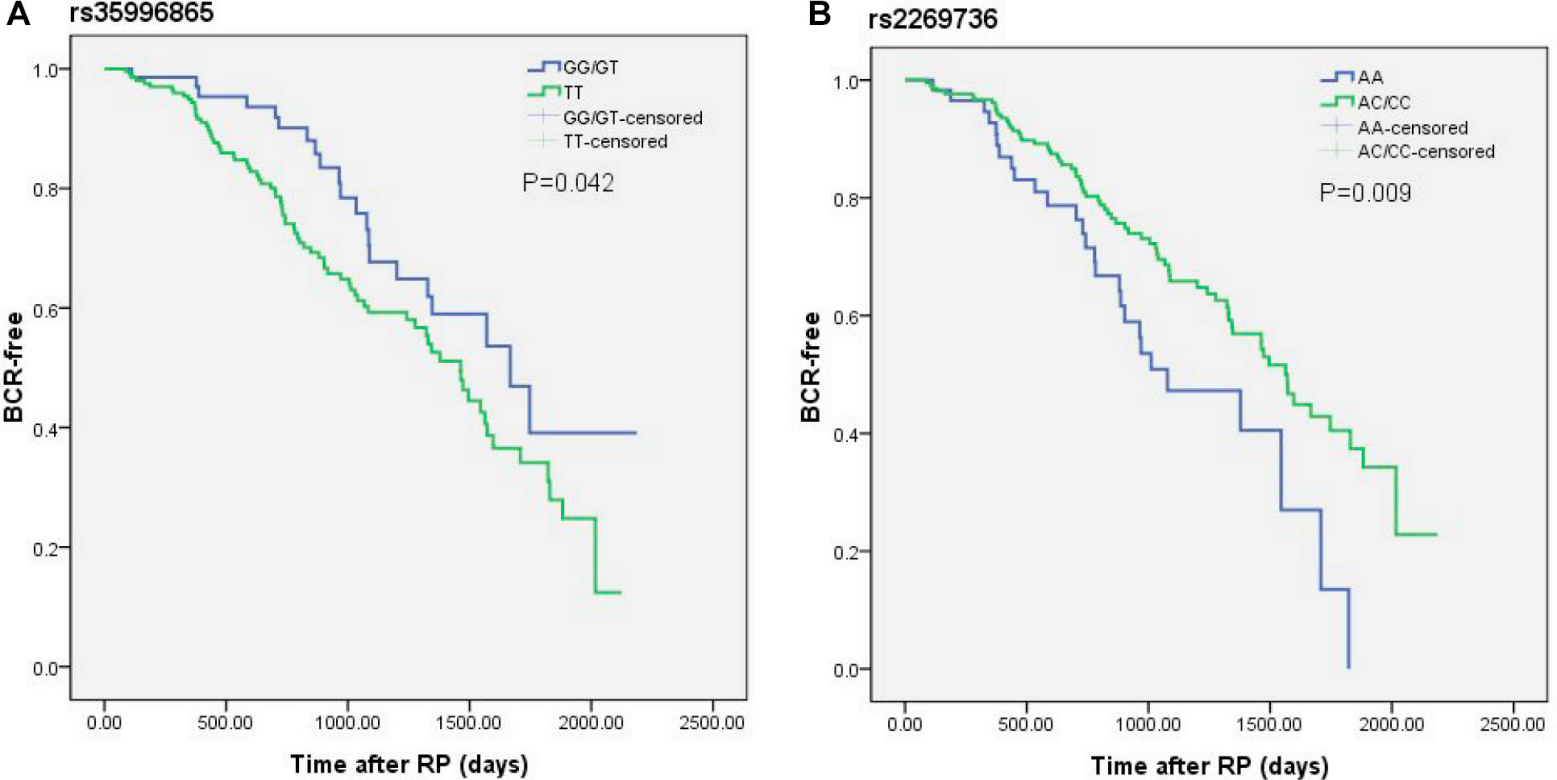
Kaplan-Meier curves of biochemical recurrence for (**A**) rs35996865 and (**B**) rs2269736 in a cohort of 289 PCa patients after radical prostatectomy. Log-rank (LR) *P* values are shown in each frame.

Kaplan-Meier curves were used to study biochemical recurrence-free survival across known risk factors for BCR. As expected, the risk of BCR was greatly linked with clinico-pathological characteristics (Supplementary Figure 1). Generally speaking, patients with higher PSA levels, higher Gleason score, nodal invasion and positive surgical margin were more susceptible to BCR. As shown in Figure 1, it was worth noting that RhoA rs2269736 and ROCK1 rs35996865 were in relation to hazard of BCR. Subjects with rs2269736 wild allele and rs35996865 mutant allele undertook higher risk of BCR, which indicated genetic factors might be promising biomarkers in predicting BCR. However, after Bonferroni correction with *P* value adjusted as 0.05/3, the relationship between rs35996865 and BCR was invalid.

We further performed multivariate Cox proportional hazard analysis for BCR of PCa patients. Table 5 presented the results of multivariate analysis. Interestingly, we finally found that carriers of rs2269736 AA genotype had shorter BCR survival than carriers of AG/GG genotypes (HR = 0.60, 95% CI = 0.38-0.95, *P* = 0.030). Simultaneously, we combined rs2269736 with rs35996865 and observed meaningful difference between BCR survival of patients with number of risk allele. The results revealed individuals with more risk alleles had poorer prognoses. Unfortunately, the results were insignificant after Bonferroni correction.

**Table 5:** Genotypes frequencies of all genetic variants and their association with biochemical recurrence

Gene	SNPs	genotype	No BCR patients	BCR patients	BCR rate (%)	Cox multivariate analysis^a^	
			(*N* = 189)	(*N* = 100)		HR (95% CI)	*P*^b^
RhoA	rs2410	AA	41	23	35.9	1.00 (reference)	0.732
		AC/CC	148 (92/56)	77 (47/30)	34.2	0.92 (0.56–1.50)	
	rs2625955	AA	79	37	31.9	1.00 (reference)	0.890
		AC/CC	110 (81/29)	63 (52/11)	36.4	1.03 (0.68–1.57)	
	rs2269736	AA	35	27	43.5	1.00 (reference)	**0.030**
		AG/GG	154 (96/58)	73 (44/29)	32.2	**0.60 (0.38–0.95)**	
ROCK1	rs11874761	GG	141	80	36.2	1.00 (reference)	0.236
		AA/AG	48 (3/45)	20 (2/18)	29.4	0.74 (0.46–1.22)	
	rs35996865	TT	137	79	36.6	1.00 (reference)	0.129
		GG/GT	52 (6/46)	21 (2/19)	28.8	0.68 (0.42–1.12)	
	rs8089974	TT	141	77	35.3	1.00 (reference)	0.890
		GG/GT	48 (2/46)	23 (1/22)	32.4	0.97 (0.60–1.56)	
Risk alleles	rs2269736+	0-1	16	2	11.1	1.00 (reference)	**0.045**
		rs35996865	2-4	173	98	36.2	**4.25 (1.03–17.46)**

Bold values indicated significant differences between two groups.

BCR = biochemical recurrence; HR = hazard ratio; CI = confidence interval

^a^adjusted for PSA at diagnosis, Gleason score, stage, age at diagnosis, bmi, smoking status, surgical margin status, and nodal invasion status.

^b^All *P* values were Bonferroni corrected, and statistical significance was set at *P* < 0.01667 (0.05/3, in tSNPs analysis ) or *P* < 0.00556 (0.05/9, in combined analysis).

## DISCUSSION

Recently, the RhoA/ROCK1 signaling pathway has received considerable attention for its involvement in tumor formation and progression. To date, the level of RhoA expression was proved to increase in skin, gastric, testicular cancer and so on [22–24]. Likewise, elevated RhoA expression was found to correspond to poor prognosis and high recurrence rates in particular cancers [25, 26]. As a pivotal effector of RhoA, ROCK1 was reported to express aberrantly in a variety of tumors as well [27, 28]. In view of crucial functions of RhoA/ROCK1 pathway in tumorigenesis, many scholars focused on its role in PCa and confirmed their close linkage. However, what mechanism behind this association was still not well-known. In particular, whether SNPs in RhoA and ROCK1 genes might play a role in tumorigenesis has not been clarified before. To our best knowledge, this is the first report of an association of genetic variants in RhoA and ROCK1 genes with development and BCR in PCa patients.

In this study, we observed subjects carrying mutant C allele of rs2410 were liable to develop PCa while those with mutant G allele of rs2269736 and mutant T allele of rs35996865 were relatively safe. Some possible explanations may account for these results. Angiogenesis is regarded as one of the hallmarks of tumor initiation and progression, which depends on the homeostatic balance between pro-angiogenic and anti-angiogenic factors [29]. Tumor cells can secrete pro-angiogenic factors and promote vascularization, which further leads to an increase in microvessel density (MVD) [30]. Several articles have manifested MVD is greater in PCa than benign prostatic hyperplasia [31]. Similarly, increased MVD was clarified to be correlated with PCa occurrence and differentiation [32]. Coincidentally, the RhoA/ROCK1 signaling pathway was reported to play a role in each key step of angiogenesis [33]. A study published in PNAS surveyed endothelial cells (ECs) of transgenic mice bearing prostate adenocarcinoma and indicated that high level of baseline activity of RhoA and ROCK1 might give rise to aberrant behaviors of prostate cancer ECs [34]. Furthermore, other researches demonstrated that inhibitors of RhoA/ROCK pathway could effectively suppress angiogenesis [35]. Thereby, we considered SNPs in RhoA and ROCK1 may lead to altered angiogenesis, and eventually presented different PCa predisposition. In addition, studies indicated hypoxia promoted tumor cell motility via RhoA and ROCK1 signaling pathways [36]. It is acknowledged that cell in tumor tissues can secret many pro-angiogenic growth factors such as VEGF in response to hypoxia [37]. VEGF may activate RhoA by guanine nucleotide exchange factors (GEFs) and further regulate downstream effecter ROCK1. In consequence, we speculated SNPs in RhoA were probable to influence catalytic efficiency of GEFs and ROCK1 polymorphisms might change the affinity towards RhoA. Subsequent researches on relevant pathways and cellular functions are needed.

Furthermore, we combined genotypes of two polymorphism loci in RhoA and observed a protective effect of CCGG genotype. Theoretically, subjects carrying CCAA joint genotype were apt to suffer from PCa while those with AAGG genotype were safest. However, we only perceived that AAGG joint genotype had a boundary protective effect. We supposed that different genetic variations had discrepant capacities or powers to affect PCa susceptibility. Apparently, rs2269736 mutant allele had stronger protective effect than rs2410 wild allele. Besides, relatively small sample size in each joint genotype could not be overlooked. To reduce interference of small sample size, we combined risk alleles and found only individuals with 3 or 4 risk alleles were at high risk of PCa. We conjectured that individuals with 1 or 2 rs2410 risk alleles carried rs2269736 mutant alleles at the same time, which counteracted risk effect of rs2410 mutant alleles and further caused those people with less than 2 risk alleles were not associated with increased hazard of PCa. We thought these outcomes were consistent with results of joint genotype analysis. Combined analysis may provide a more comprehensive prediction of genetic susceptibility. Nevertheless, it is universally accepted that PCa is a complex malignancy arose from multifactor. Results of further subgroup analysis suggested that single loci polymorphisms and the combined effect were more pronounced in some specific population groups, which indicated that genetic effects on PCa susceptibility may be interfered by age, physical condition and environment exposure. Further study with larger sample size should be conducted to draw more convinced conclusion.

As we mentioned above, the RhoA/ROCK signaling pathway was closely related with MVD, angiogenesis and VEGF. Bono AV and his colleagues declared that MVD counts were associated with PCa progression and might potentially predict outcome in patients undergoing RP [38]. Another work showed that angiogenesis was also linked with PCa progression after RP [39]. Besides, Peyromaure M et al. compared non-relapsed patient after RP with those developed metastases and found the expression of VEGF was obviously different between two groups [40]. Taking these observations into consideration, we assumed that genetic variants in RhoA and ROCK1 genes stood a chance to participate in PCa progression and recurrence. Oddly, we did not observe any meaningful correlation between clincopathologic parameters with single loci polymorphism or combined risk alleles. It has been proposed that BCR risk after RP was greatly influenced by clinical and pathologic characteristics in European and Asian populations [41]. Cotignola J et al. subsequently affirmed similar conclusions as well in Argentinean [42]. As expected, the results in our present study were in accordance with previous studies. In the meanwhile, we also discovered RhoA rs2269736 and ROCK1 rs35996865 were connected with the time to BCR. The association between rs2269736 and BCR was consistently observed when using relevant confounders such as age, PSA, Gleason score and so on as covariates in multivariate Cox analysis, indicative of the strength of their relationship and the potential of rs2269736 to be a biomarker. In fact, association of genetic mutations in RhoA/ROCK1 pathway with BCR after RP is not unexpected. It was established that the RhoA/ROCK signaling pathway regulated abundant tumor cellular processes ranging from proliferation, growth and invasion to cytoskeletal remodeling and gene expression [43].

Campa M et al. reported identification of functional membrane androgen receptors (mAR) and found its activation could induce rapid actin cytoskeleton reorganization and increased secretion of PSA. Recently, their research team indicated that RhoA and ROCK1 were major mAR effectors adjusting actin reorganization and apoptosis in PCa cells [21]. Accordingly, it was logical to surmise that functional genetic variants in RhoA and ROCK1 were likely to result in different activity of RhoA/ROCK1 and affect functions of PCa cells, which led to BCR in PCa patients. Noteworthily, an article put forwards a novel mechanism of androgen action in PCa which was mediated by action of the transcription factor serum response factor (SRF). When compared with androgen target genes, the authors found that the expressions of androgen-dependent and SRF-dependent genes were associated robustly with BCR [44]. Latter study demonstrated that the RhoA/ROCK1 pathway mediated androgen-responsiveness of a majority of SRF target genes and interfered with clinically relevant androgen action in PCa [45]. Based on aforementioned findings, we hypothesized the association between genetic variants in RhoA/ROCK1 pathway and disparate prognosis of PCa patients might attribute to significant roles of RhoA/ROCK1 in androgen action. Individuals carrying risk alleles were possibly more sensitive to androgen stimulation and undertook higher risk of BCR. However, additional researches refer to underlying biologic mechanisms driving the positive associations of genetic variants in the RhoA/ROCK1 pathway with BCR are demanded to verify our hypotheses.

Remarkably, Bonferroni correction was utilized in our study. As strict correction method, it is one of the most important methods used to address false discovery rates resulting from multiple testing. The Bonferroni correction acts as conservative method and adjusts the value of alpha according to the number of tests performed. Moreover, truly significant differences may be deemed non-significant because of type II errors [46]. Accordingly, it could reduce false positive results, but might increase false negative results. However, as preliminary study to explore genetic variants of RhoA and ROCK1 genes, we would like to find more connections as far as possible. So, we should also give consideration to traditional *P* value as 0.05. Though, no statistical significance was detected in some new findings with Bonferroni correction, further studies with larger sample size were required to confirm such results.

Taken together, the results in our present study provided new insight into genetic variants in RhoA/ROCK1 signaling pathway and their function in prostate cancer. We believed our findings were helpful to clinical diagnosis and prediction of prostate cancer. Also, our study gave rise to the idea that meaningful SNPs in RhoA/ROCK1 pathway and some combined genotypes might be favorable biomarkers for PCa prognosis, which guided clinicians to formulate individualized therapy regimen. However, some limitations of this study should be noted. Firstly, to apply new markers in the clinical practice needs complicated steps and diverse validation analyses. More studies on expression and activity of the RhoA/ROCK1 pathway in subjects with different genotypes are indispensible. Secondly, our findings merit further evaluation in larger series of PCa patients from different regions, taking into account that prostate cancer is a heterogeneous illness with multiple confounders. Moreover, Bonferroni correction was utilized in our study. Though, no statistical significance was detected in some genotypes or subgroups with Bonferroni correction, further studies with larger sample size were required to confirm such results. Last but not the least, lack of detailed data of survival and other risk factors from patients limits further in-depth investigation.

To sum up, to our knowledge, the current study firstly provided evidences to certify genetic variants in the RhoA/ROCK1 pathway, especially rs2269736 and combined risk alleles may be promising novel predictors to forecast development, progression and prognosis of prostate cancer. Undoubtedly, the conclusions may broaden our horizons in the biological basis of carcinogenesis of prostate cancer and be beneficial to offer patients reasonable treatment.

## MATERIALS AND METHODS

### Study population

We prospectively recruited 830 patients with histopathologically confirmed PCa and a group of 869 cancer free controls who sought routine outpatient care between September 2003 and January 2013 from The First Hospital of Nanjing Medical University, Nanjing, China. All these subjects were genetically unrelated Han Chinese. All controls had no history of other cancers and were matched on age to the cases. Before recruitment, a standard questionnaire was administered through face-to-face interviews by well-trained interviewers to collect demographic data, clinical data and related factors. The definitions of subgroups were described in our previous paper [47]. The clinical stage was divided into the localized and advanced cancer (localized: T1–2N0M0; advanced: T3-4NxMx or TxN1Mx or TxNxM1) based on the tumor-node-metastasis staging system. The Gleason score was estimated by pathologists working at the hospital. Patients were categorized into three groups according to their serum PSA value: PSA ≤ 10 ng/ml, PSA 10–20 ng/ml and PSA > 20 ng/ml. Each subject donated 5 ml of venous blood after written informed consent. For the biochemical recurrence analysis, a total of 289 PCa cases enrolled in our ongoing cohort study. All patients underwent radical prostatectomy as their primary therapeutic strategy and had complete follow-up data. Follow-up and maintenance of updated medical records were performed by trained urologists. The research protocol was approved by the institutional review board of Nanjing Medical University and the study was carried out in accordance with the nationally approved guidelines. All patients who agreed to participate in the study signed a written informed consent.

### SNP selection, tSNPs identification and genotyping

We chose 3 potentially functional tSNPs in RhoA gene (rs2410, rs2625955 and rs2269736) and 3 tSNPs in ROCK1 gene (rs11874761, rs35996865 and rs8089974) according to HapMap data (HapMap Data Rel 24/Phase II, Nov08, on NCBI B36 assembly, dbSNP b126) and the Haploview 4.2 software (Cambridge, MA, USA) (Supplementary Figure 2). All selected SNPs were located in the 5′ flanking regions, 5′ untranslated region (UTR), 3′ UTR, or coding regions with amino acid changes and minor allele frequency (MAF) of each polymorphism was greater than 5% in Chinese population. When some of the SNPs were in complete linkage disequilibrium (r^2^ = 1), only one SNP was selected for genotyping. Genomic DNA was extracted from the peripheral blood by proteinase K digestion and phenol–chloroform extraction. Before genotyping, we conducted DNA quality control and made sure that all DNA samples were qualified. Genotyping was performed with the TaqMan SNP Genotyping Assay using the 384-well ABI 7900HT real-time PCR system (Applied Biosystems, Foster City, CA, USA). The tSNPs information, sequences of primers and probes of each SNP were available as requested. Negative controls were included in each plate to ensure accuracy of the genotyping. Two investigators conducted genotyping independently in a blinded manner. Finally, about 10% of the DNA samples were randomly chose for repeated genotyping for confirmation, and the results were 100% concordant.

### Statistical analysis

Deviation of genotype distribution from the Hardy–Weinberg equilibrium (HWE) for all polymorphisms among the controls was tested by a goodness-of-fit chi-square test. Differences in the distributions of demographic characteristics, selected variables, and frequencies of genotypes between cases and controls were analyzed using the Student's t-test (for continuous variables) or chi-square test (for categorical variables). The associations between polymorphisms and risk of PCa were estimated by computing odds ratios (ORs) and 95% confidence intervals (CIs) from unconditional logistic regression analysis with the adjustment for possible confounders. Biochemical recurrence was defined as a rise in serum PSA level 0.2 ng/ml after RP. To study BCR-free survival, time was calculated from date of RP to date of BCR or last follow up. The different recurrence times according to demographic characteristics, clinical features, and RhoA/ROCK1 polymorphisms were evaluated by Kaplan–Meier method and compared by the log-rank test. Univariate and multivariate analyses were performed using Cox proportional hazard models to determine the association between polymorphisms and PCa biochemical recurrence and to estimate adjusted Hazard Ratios (HRs) and 95% CIs with adjustment for possible confounders. D^2^ and r^2^ value for linkage disequilibrium (LD) between the two polymorphisms were estimated by the Haploview software version 4.2. The Bonferroni correction is one of the most frequently-used methods, and aims to address false discovery rates caused by multiple testing. Accordingly, all *P* values were Bonferroni corrected, and statistical significance was set at *P* < 0.01667 (0.05/3, in genotype and allele analysis) or *P* < 0.00556 (0.05/9, in combined analysis). All statistical tests were two-sided. All the statistical analyses were performed with the Stata software (version 12.1; StataCorp LP, College Station, TX, USA) or SPSS version 17.0 for Windows (SPSS Inc., Chicago, IL, USA).

## SUPPLEMENTARY MATERIALS FIGURES AND TABLES






