# Supplementary material for: Genetic variants in RhoA and ROCK1 genes are associated with the development, progression and prognosis of prostate cancer
Source: Oncotarget. 2017 Feb 8;8(12):19298–309. doi: 10.18632/oncotarget.15197 (PMC5386685; doi:10.18632/oncotarget.15197)
Supplement: Supplementary file 1 [file oncotarget-08-19298-s001.pdf]

Genetic variants in RhoA and ROCK1 genes are associated with the development, progression and prognosis of prostate cancer

Supplementary Materials

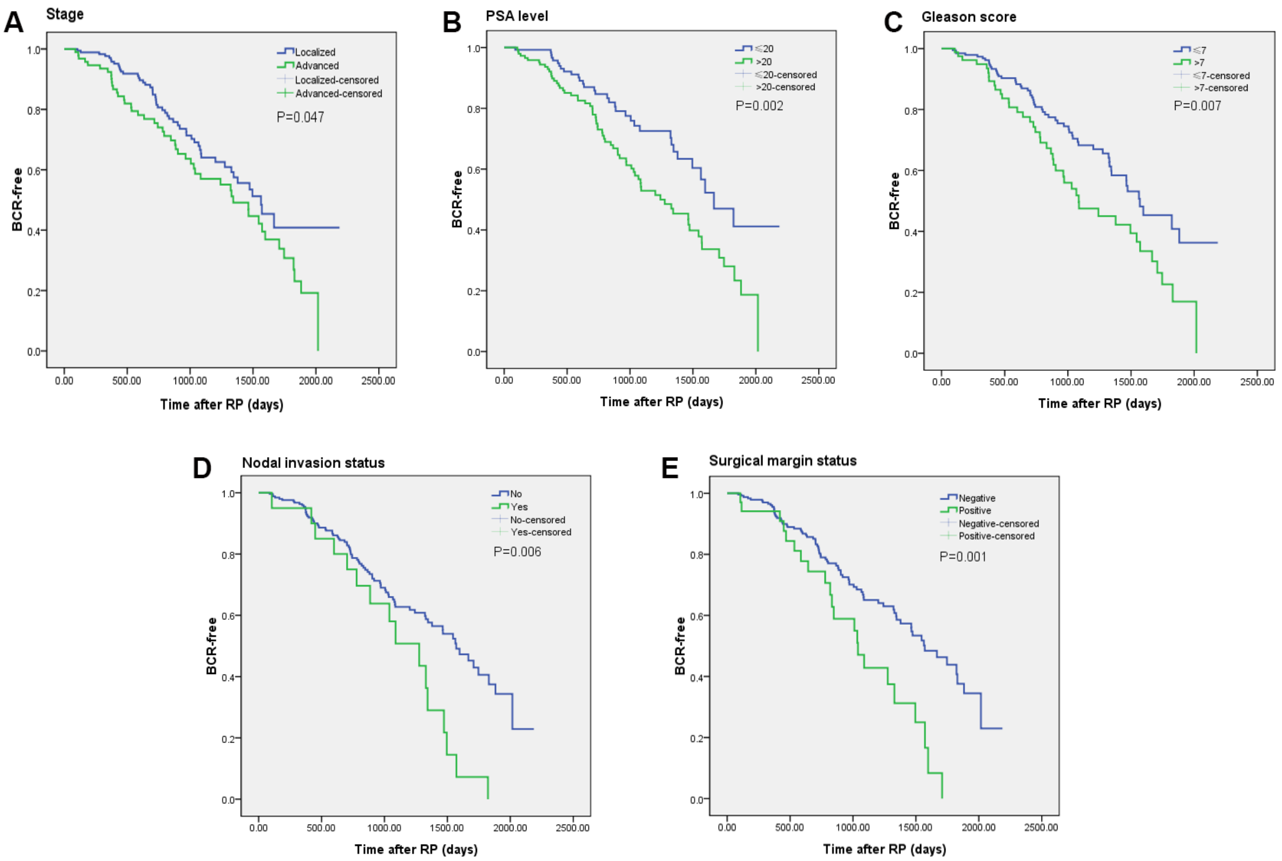

Supplementary Figure 1: Kaplan-Meier curves of biochemical recurrence for (**A**) stage, (**B**) PSA level, (**C**) Gleason score, (**D**) Nodal invasion status and (**E**) Surgical margin status in a cohort of 289 PCa patients after radical prostatectomy. Log-rank (LR) *P* values are shown in each frame.

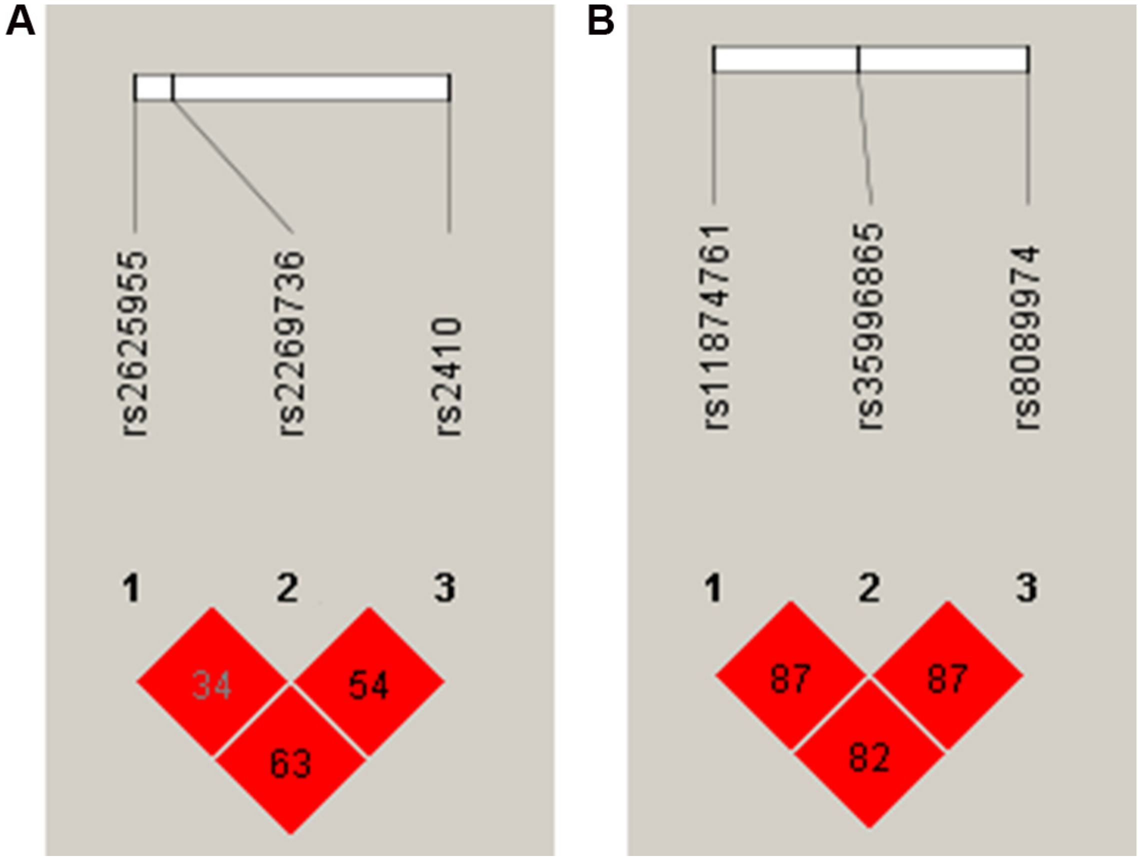

Supplementary Figure 2: Haplotype block map for RhoA tSNPs and ROCK1 tSNPs. (**A**) RhoA tSNPs includes rs2625955, rs2269736 and rs2410, (**B**) ROCK1 tSNPs includes rs11874761, rs35996865 and 8089974. The linkage magnitude between polymorphisms was qualified by the D′ and logarithm of odds (LOD). The linkage plot was displayed according to the standard color scheme.

Supplementary Table 1A: Stratification analysis of rs2410 and PCa risk. See Supplementary_Table_1A

Supplementary Table 1B: Stratification analysis of rs2625955 and PCa risk. See Supplementary_Table_1B

Supplementary Table 1C: Stratification analysis of rs2269736 and PCa risk. See Supplementary_Table_1C

Supplementary Table 1D: Stratification analysis of rs11874761 and PCa risk. See Supplementary_Table_1D

Supplementary Table 1E: Stratification analysis of rs35996865 and PCa risk. See Supplementary_Table_1E

Supplementary Table 1F: Stratification analysis of rs8089974 and PCa risk. See Supplementary_Table_1F

Supplementary Table 2A: Association between rs2410 and clinicopathologic parameters of PCa. See Supplementary_Table_2A

Supplementary Table 2B: Association between rs2625955 and clinicopathologic parameters of PCa. See Supplementary_Table_2B

Supplementary Table 2C: Association between rs2625955 and clinicopathologic parameters of PCa. See Supplementary_Table_2C

Supplementary Table 2D: Association between rs11874761 and clinicopathologic parameters of PCa. See Supplementary_Table_2D

Supplementary Table 2E: Association between rs35996865 and clinicopathologic parameters of PCa. See Supplementary_Table_2E

Supplementary Table 2F: Association between rs8089974 and clinicopathologic parameters of PCa. See Supplementary_Table_2F.

Supplementary Table 2G: Association between number of risk alleles and clinicopathologic parameters of PCa. See Supplementary_Table_2G

Supplementary Table 3: Clinico-pathological characteristics of study populations included in biochemical recurrence analysis

Variables	*N*	Percentage
**Total cases**	289	100
**Biochemical recurrence**		
**No**	189	65.4
**Yes**	100	34.6
**Age (median ± SD)**	71	±7.58
**PSA at diagnosis (ng/ml)**		
**≤ 10**	134	46.4
**> 10**	155	53.6
**Gleason score**		
**≤ 7**	203	70.2
**> 7**	86	29.8
**Stage**		
**localized**	190	65.7
**Advanced**	99	34.3
**Nodal invasion**		
**No**	225	77.9
**Yes**	64	22.1
**Margin status**		
**Negative**	227	78.5
**Positive**	62	21.5
**Smoke status**		
**Never**	121	41.9
**Ever**	168	58.1
**Drinking status**		
**Never**	204	70.6
**Ever**	85	29.4

SD = standard deviation; PSA = prostate-specific antigen.
