# Supplementary material for: Genetic variants in RhoA and ROCK1 genes are associated with the development, progression and prognosis of prostate cancer
Source: Oncotarget. 2017 Feb 8;8(12):19298–309. doi: 10.18632/oncotarget.15197 (PMC5386685; doi:10.18632/oncotarget.15197)
Supplement: Supplementary file 2 [file oncotarget-08-19298-s002.docx]

| **Supplementary Table 2A.** Association between rs2410 and clinicopathologic parameters of PCa | | | | | | | |
| --- | --- | --- | --- | --- | --- | --- | --- |
| rs2410 | case | AA | | AC+CC | | P^b^ | Adjusted OR(95%CI)^a^ |
| Variable |  | n | % | n | % |  | AC+CC vs. AA |
| **Clinical stage** | | | | | | 0.708 |  |
| **Localized** | 498 | 120 | 24.1 | 378 | 75.9 |  | 1.00(reference) |
| **Advanced** | 332 | 79 | 23.8 | 253 | 76.2 |  | 1.02(0.90-1.16) |
| **Gleason score** | | | | | |  |  |
| **<7** | 233 | 53 | 22.8 | 180 | 77.2 |  | 1.00(reference) |
| **=7** | 302 | 73 | 24.2 | 229 | 75.8 | 0.887 | 0.99(0.85-1.15) |
| **>7** | 295 | 73 | 24.8 | 222 | 75.2 | 0697 | 1.03(0.89-1.20) |
| **PSA(ng/ml)** | | | | | |  |  |
| **≤10** | 175 | 42 | 24.0 | 131 | 76.0 |  | 1.00(reference) |
| **10-20** | 172 | 40 | 23.3 | 132 | 76.7 | 0.616 | 1.05(0.87-1.26) |
| **>20** | 483 | 117 | 24.3 | 366 | 75.7 | 0.450 | 0.94(0.81-1.10) |

^a^Adjusted for age, bmi, pack-years of smoking, drinking status, tea drinking, hypertension and diabetes in logistic regression model.

^b^All P values were Bonferroni corrected, and statistical significance was set at p<0.01667 (0.05/3).

**Supplementary Table 2B.** Association between rs2625955 and clinicopathologic parameters of PCa

| rs2625955 | case | AA | | AC+CC | | P^b^ | Adjusted OR(95%CI)^a^ |
| --- | --- | --- | --- | --- | --- | --- | --- |
| Variable |  | n | % | n | % |  | AC+CC vs. AA |
| **Clinical stage** | | | | | | 0.526 |  |
| **Localized** | 498 | 193 | 38.8 | 305 | 61.2 |  | 1.00(reference) |
| **Advanced** | 332 | 132 | 39.8 | 200 | 60.2 |  | 1.05(0.91-1.20) |
| **Gleason score** | | | | | |  |  |
| **<7** | 233 | 97 | 41.6 | 136 | 58.4 |  | 1.00(reference) |
| **=7** | 302 | 111 | 36.8 | 191 | 63.2 | 0.087 | 1.17(0.98-1.39) |
| **>7** | 295 | 117 | 39.7 | 178 | 60.3 | 0.383 | 1.08(0.91-1.29) |
| **PSA(ng/ml)** | | | | | |  |  |
| **≤10** | 175 | 75 | 42.9 | 100 | 57.1 |  | 1.00(reference) |
| **10-20** | 172 | 63 | 36.7 | 109 | 63.3 | 0.917 | 1.01(0.81-1.26) |
| **>20** | 483 | 187 | 38.7 | 296 | 61.3 | 0.414 | 1.08(0.90-1.28) |

^a^Adjusted for age, bmi, pack-years of smoking, drinking status, tea drinking, hypertension and diabetes in logistic regression model.

^b^All P values were Bonferroni corrected, and statistical significance was set at p<0.01667 (0.05/3).

**Supplementary Table 2C.** Association between rs2625955 and clinicopathologic parameters of PCa

| rs2269736 | case | AA | | AG+GG | | P^b^ | Adjusted OR(95%CI)^a^ |
| --- | --- | --- | --- | --- | --- | --- | --- |
| Variable |  | n | % | n | % |  | AG+GG vs. AA |
| **Clinical stage** | | | | | | 0.473 |  |
| **Localized** |  | 107 | 21.5 | 391 | 78.5 |  | 1.00(reference) |
| **Advanced** |  | 79 | 23.8 | 253 | 76.2 |  | 1.05(0.92-1.19) |
| **Gleason score** | | | | | |  |  |
| **<7** | 233 | 55 | 23.6 | 178 | 76.4 |  | 1.00(reference) |
| **=7** | 302 | 66 | 21.9 | 236 | 78.1 | 0.087 | 1.17(0.98-1.39) |
| **>7** | 295 | 65 | 22.0 | 230 | 78.0 | 0.383 | 1.08(0.91-1.29) |
| **PSA(ng/ml)** | | | | | |  |  |
| **≤10** | 175 | 45 | 25.7 | 130 | 74.3 |  | 1.00(reference) |
| **10-20** | 172 | 34 | 19.8 | 138 | 80.2 | 0.763 | 0.97(0.80-1.18) |
| **>20** | 483 | 107 | 22.2 | 376 | 77.8 | 0.756 | 0.98(0.84-1.14) |

^a^Adjusted for age, bmi, pack-years of smoking, drinking status, tea drinking, hypertension and diabetes in logistic regression model.

^b^All P values were Bonferroni corrected, and statistical significance was set at p<0.01667 (0.05/3).

**Supplementary Table 2D.** Association between rs11874761 and clinicopathologic parameters of PCa

| rs11874761 | case | AA | | AG+GG | | P^b^ | Adjusted OR(95%CI) ^a^ |
| --- | --- | --- | --- | --- | --- | --- | --- |
| Variable |  | n | % | n | % |  | AG+GG vs. AA |
| **Clinical stage** | | | | | | 0.214 |  |
| **Localized** | 498 | 10 | 2.0 | 488 | 98.0 |  | 1.00(reference) |
| **Advanced** | 332 | 2 | 0.6 | 330 | 99.4 |  | 1.11(0.94-1.31) |
| **Gleason score** | | | | | |  |  |
| **<7** | 233 | 2 | 0.9 | 231 | 99.1 |  | 1.00(reference) |
| **=7** | 302 | 6 | 2.0 | 296 | 98.0 | 0.229 | 0.88(0.72-1.08) |
| **>7** | 295 | 4 | 1.4 | 291 | 98.6 | 0.598 | 0.95(0.77-1.16) |
| **PSA(ng/ml)** | | | | | |  |  |
| **≤10** | 175 | 2 | 1.1 | 173 | 98.9 |  | 1.00(reference) |
| **10-20** | 172 | 2 | 1.2 | 170 | 98.8 | 0.100 | 1.24(0.96-1.59) |
| **>20** | 483 | 8 | 1.7 | 475 | 98.3 | 0.123 | 1.17(0.96-1.42) |

^a^Adjusted for age, bmi, pack-years of smoking, drinking status, tea drinking, hypertension and diabetes in logistic regression model.

^b^All P values were Bonferroni corrected, and statistical significance was set at p<0.01667 (0.05/3).

**Supplementary Table 2E.** Association between rs35996865 and clinicopathologic parameters of PCa

| rs35996865 | case | GG | | GT+TT | | P^b^ | Adjusted OR(95%CI) ^a^ |
| --- | --- | --- | --- | --- | --- | --- | --- |
| Variable |  | n | % | n | % |  | GT+TT vs. TT |
| **Clinical stage** | | | | | | 0.566 |  |
| **Localized** | 498 | 9 | 1.8 | 489 | 98.2 |  | 1.00(reference) |
| **Advanced** | 332 | 7 | 2.1 | 325 | 97.9 |  | 1.05(0.89-1.23) |
| **Gleason score** | | | | | |  |  |
| **<7** | 233 | 3 | 1.3 | 230 | 98.7 |  | 1.00(reference) |
| **=7** | 302 | 6 | 2.0 | 296 | 98.0 | 0.360 | 0.91(0.75-1.11) |
| **>7** | 295 | 7 | 2.4 | 288 | 97.6 | 0.286 | 0.90(0.74-1.09) |
| **PSA(ng/ml)** | | | | | |  |  |
| **≤10** | 175 | 3 | 1.7 | 172 | 98.3 |  | 1.00(reference) |
| **10-20** | 172 | 3 | 1.7 | 169 | 98.3 | 0.133 | 1.21(0.94-1.54) |
| **>20** | 483 | 10 | 2.1 | 473 | 97.9 | 0.311 | 1.10(0.91-1.33) |

^a^Adjusted for age, bmi, pack-years of smoking, drinking status, tea drinking, hypertension and diabetes in logistic regression model.

^b^All P values were Bonferroni corrected, and statistical significance was set at p<0.01667 (0.05/3).

**Supplementary Table 2F.** Association between rs8089974 and clinicopathologic parameters of PCa

| rs8089974 | case | GG | | GT+TT | | P^b^ | Adjusted OR(95%CI) ^a^ |
| --- | --- | --- | --- | --- | --- | --- | --- |
| Variable |  | n | % | n | % |  | GT+TT vs. GG |
| **Clinical stage** | | | | | | 0.437 |  |
| **Localized** | 498 | 8 | 1.6 | 490 | 98.4 |  | 1.00(reference) |
| **Advanced** | 332 | 0 | 0 | 332 | 100 |  | 1.07(0.90-1.26) |
| **Gleason score** | | | | | |  |  |
| **<7** | 233 | 3 | 1.3 | 230 | 98.7 |  | 1.00(reference) |
| **=7** | 302 | 3 | 1.0 | 299 | 99.0 | 0.807 | 0.98(0.80-1.19) |
| **>7** | 295 | 2 | 0.7 | 293 | 99.3 | 0.420 | 1.09(0.89-1.34) |
| **PSA(ng/ml)** | | | | | |  |  |
| **≤10** | 175 | 2 | 1.7 | 172 | 98.3 |  | 1.00(reference) |
| **10-20** | 172 | 1 | 0.6 | 171 | 99.4 | 0.416 | 1.11(0.86-1.43) |
| **>20** | 483 | 4 | 0.8 | 479 | 99.2 | 0.377 | 1.09(0.90-1.33) |

^a^Adjusted for age, bmi, pack-years of smoking, drinking status, tea drinking, hypertension and diabetes in logistic regression model.

^b^All P values were Bonferroni corrected, and statistical significance was set at p<0.01667 (0.05/3).

**Supplementary Table 2G.** Association between number of risk alleles and clinicopathologic parameters of PCa

| Variable | Risk alleles(0-1) | | Risk alleles(2-4) | | P^b^ | Adjusted OR (95%CI) ^a^ |
| --- | --- | --- | --- | --- | --- | --- |
|  | n | % | n | % |  |  |
| **Clinical stage** | | | | | 0.290 |  |
| **Localized** | 155 | 31.1 | 343 | 68.9 |  | 1.0(reference) |
| **Advanced** | 115 | 34.6 | 217 | 65.4 |  | 0.85(0.63-1.15) |
| **Gleason score** | | | | | 0.627 |  |
| **<7** | 70 | 30.0 | 163 | 70.0 |  | 1.0(reference) |
| **=7** | 102 | 33.8 | 200 | 66.2 |  | 0.86(0.60-1.25) |
| **>7** | 98 | 33.2 | 197 | 66.8 |  | 0.84(0.57-1.22) |
| **PSA(ng/ml)** | | | | | 0.625 |  |
| **≤10** | 54 | 30.9 | 121 | 69.1 |  | 1.0(reference) |
| **10-20** | 61 | 35.5 | 111 | 64.5 |  | 0.85(0.53-1.35) |
| **>20** | 155 | 32.1 | 328 | 67.9 |  | 0.93(0.63-1.35) |

^a^Adjusted for age, bmi, pack-years of smoking, drinking status, tea drinking, hypertension and diabetes in logistic regression model.

^b^All P values were Bonferroni corrected, and statistical significance was set at p<0.00556 (0.05/9).
