# Supplementary material for: Genetic variants in RhoA and ROCK1 genes are associated with the development, progression and prognosis of prostate cancer
Source: Oncotarget. 2017 Feb 8;8(12):19298–309. doi: 10.18632/oncotarget.15197 (PMC5386685; doi:10.18632/oncotarget.15197)
Supplement: Supplementary file 3 [file oncotarget-08-19298-s003.docx]

| **Supplementary Table 1A**. Stratification analysis of rs2410 and PCa risk. | | | | | | | |
| --- | --- | --- | --- | --- | --- | --- | --- |
| rs2410 | Case/Control | Genotypes | | | | P^b^ | Adjusted OR(95%CI)^a^ |
|  |  | CC | | AC+CC | |  |  |
| Variable |  | n | % | n | % |  | AC+CC vs. AA |
| **Total** | 830/869 | 253/221 | 30.5/25.4 | 631/636 | 76.0/73.2 | **0.016** | **1.11(1.02-1.21)** |
| **Age** | | | | | | | |
| **≤71** | 383/429 | 114/112 | 30.0/26.1 | 287/307 | 74.9/71.6 | 0.215 | 1.08(0.95-1.23) |
| **>71** | 447/440 | 139/109 | 31.1/24.8 | 344/329 | 77.0/74.8 | 0.053 | 1.12(1.00-1.27) |
| **Smoke status** | | | | | | | |
| **Never** | 325/392 | 101/99 | 31.1/25.3 | 253/277 | 77.8/70.7 | 0.050 | 1.14(1.00-1.31) |
| **Ever** | 505/477 | 152/122 | 30.3/25.6 | 378/359 | 74.9/75.3 | 0.156 | 1.09(0.97-1.22) |
| **Pack-years of smoking** | | | | | | | |
| **0-20** | 229/232 | 61/65 | 26.6/28.0 | 167/176 | 72.9/75.9 | 0.967 | 1.00(0.85-1.19) |
| **>20** | 276/245 | 91/57 | 33.0/23.3 | 211/183 | 76.4/74.7 | **0.046** | **1.18(1.00-1.38)** |
| **Drinking status** | | | | | | | |
| **Never** | 582/638 | 178/163 | 30.6/25.5 | 444/473 | 76.3/74.1 | 0.053 | 1.10(1.00-1.22) |
| **Ever** | 248/231 | 75/58 | 30.2/25.1 | 187/163 | 75.4/70.6 | 0.322 | 1.09(0.92-1.28) |
| **Tea drinking** | | | | | | | |
| **Never** | 407/383 | 136/104 | 33.4/27.2 | 315/277 | 77.4/72.3 | **0.018** | **1.16(1.03-1.31)** |
| **Ever** | 423/486 | 117/117 | 27.7/24.1 | 316/359 | 74.7/73.9 | 0.311 | 1.06(0.94-1.20) |
| **Family history of cancer** | | | | | | | |
| **No** | 639/747 | 205/191 | 32.1/25.6 | 490/545 | 76.7/73.0 | **0.009** | **1.13(1.03-1.24)** |
| **Yes** | 191/122 | 48/30 | 25.1/24.6 | 141/91 | 73.8/74.6 | 0.940 | 1.01(0.81-1.25) |
| **Hypertension** | | | | | | | |
| **No** | 614/667 | 186/170 | 30.3/25.5 | 463/487 | 73.0/75.4 | 0.055 | 1.10(1.00-1.22) |
| **Yes** | 216/202 | 67/51 | 31.0/25.3 | 168/149 | 77.8/73.8 | 0.161 | 1.13(0.95-1.35) |
| **Diabetes** | | | | | | | |
| **No** | 707/761 | 210/191 | 29.7/25.1 | 536/559 | 75.8/73.5 | **0.048** | **1.09(1.00-1.21)** |
| **Yes** | 123/108 | 43/30 | 35.0/27.8 | 95/77 | 77.2/71.3 | 0.228 | 1.16(0.91-1.46) |

Bold values indicated significant differences between two groups.

^a^Adjusted for age, bmi, pack-years of smoking, drinking status, tea drinking, hypertension and diabetes in logistic regression model.

^b^All P values were Bonferroni corrected, and statistical significance was set at p<0.01667 (0.05/3).

**Supplementary Table 1B**. Stratification analysis of rs2625955 and PCa risk.

| rs2625955 | Case/Control | Genotypes | | | | P* | Adjusted OR(95%CI) ^a^ |
| --- | --- | --- | --- | --- | --- | --- | --- |
|  |  | CC | | AC+CC | |  |  |
| Variable |  | n | % | n | % |  | AC+CC vs.AA |
| **Total** | 830/869 | 129/127 | 15.5/14.6 | 505/521 | 60.8/59.9 | 0.806 | 1.01(0.92-1.12) |
| **Age** | | | | | | | |
| **≤71** | 383/429 | 60/72 | 15.7/16.8 | 234/259 | 61.1/60.4 | 0.703 | 0.97(0.84-1.12) |
| **>71** | 447/440 | 69/55 | 15.4/12.5 | 271/262 | 60.6/59.5 | 0.422 | 1.06(0.92-1.22) |
| **Smoke status** | | | | | | | |
| **Never** | 325/392 | 52/53 | 16.0/13.5 | 198/216 | 60.9/55.1 | 0.133 | 1.12(0.97-1.31) |
| **Ever** | 505/477 | 77/74 | 15.2/15.5 | 307/305 | 60.8/63.9 | 0.361 | 0.94(0.83-1.07) |
| **Pack-years of smoking** | | | | | | | |
| **0-20** | 229/232 | 37/31 | 16.2/13.4 | 149/138 | 65.1/59.5 | 0.582 | 1.06(0.87-1.28) |
| **>20** | 276/245 | 40/43 | 14.5/17.6 | 158/167 | 57.2/68.2 | 0.069 | 0.85(0.71-1.01) |
| **Drinking status** | | | | | | | |
| **Never** | 582/638 | 89/95 | 15.3/14.9 | 349/382 | 60.0/59.9 | 0.753 | 0.98(0.87-1.10) |
| **Ever** | 248/231 | 40/32 | 16.1/13.9 | 156/139 | 63.0/60.1 | 0.261 | 1.11(0.92-1.35) |
| **Tea drinking** | | | | | | | |
| **Never** | 407/383 | 55/59 | 13.5/15.4 | 236/238 | 58.0/62.1 | 0.205 | 0.91(0.79-1.05) |
| **Ever** | 423/486 | 74/68 | 17.5/14.0 | 269/283 | 63.6/58.2 | 0.141 | 1.10(0.97-1.26) |
| **Family history of cancer** | | | | | | | |
| **No** | 639/747 | 97/106 | 15.2/14.2 | 378/443 | 59.2/59.3 | 0.785 | 1.02(0.91-1.13) |
| **Yes** | 191/122 | 32/21 | 16.8/17.2 | 127/78 | 66.5/63.9 | 0.976 | 1.00(0.79-1.26) |
| **Hypertension** | | | | | | | |
| **No** | 614/667 | 96/92 | 15.6/13.8 | 382/399 | 62.2/59.8 | 0.427 | 1.05(0.93-1.17) |
| **Yes** | 216/202 | 33/35 | 15.3/17.3 | 123/122 | 56.9/60.4 | 0.337 | 0.91(0.75-1.10) |
| **Diabetes** | | | | | | | |
| **No** | 707/761 | 107/108 | 15.1/14.2 | 434/453 | 61.4/59.5 | 0.648 | 1.03(0.92-1.14) |
| **Yes** | 123/108 | 22/19 | 17.9/17.6 | 71/68 | 57.7/63.0 | 0.474 | 0.91(0.70-1.18) |

^a^Adjusted for age, bmi, pack-years of smoking, drinking status, tea drinking, hypertension and diabetes in logistic regression model.

**Supplementary Table 1C**. Stratification analysis of rs2269736 and PCa risk.

| rs2269736 | Case/Control | Genotypes | | | | P* | Adjusted OR(95%CI) ^a^ |
| --- | --- | --- | --- | --- | --- | --- | --- |
|  |  | GG | | AG+GG | |  |  |
| Variable |  | n | % | n | % |  | AG+GG vs.AA |
| **Total** | 830/869 | 245/349 | 29.5/40.2 | 644/753 | 77.6/86.7 | **<0.001** | **0.79(0.72-0.86)** |
| **Age** | | | | | | | |
| **≤71** | 383/429 | 107/177 | 27.9/41.3 | 291/375 | 76.0/87.4 | **<0.001** | **0.74(0.65-0.84)** |
| **>71** | 447/440 | 138/172 | 30.1/39.1 | 353/378 | 79.0/86.0 | **0.003** | **0.83(0.74-0.94)** |
| **Smoke status** | | | | | | | |
| **Never** | 325/392 | 106/150 | 32.6/38.3 | 262/340 | 80.6/86.7 | 0.059 | 0.88(0.77-1.00) |
| **Ever** | 505/477 | 139/199 | 27.5/41.7 | 382/413 | 75.6/86.6 | **<0.001** | **0.73(0.65-0.81)** |
| **Pack-years of smoking** | | | | | | | |
| **0-20** | 229/232 | 72/86 | 31.4/37.0 | 176/195 | 76.9/84.0 | **0.048** | **0.85(0.72-0.99)** |
| **>20** | 276/245 | 67/113 | 24.3/46.1 | 206/218 | 74.6/89.0 | **<0.001** | **0.62(0.53-0.73)** |
| **Drinking status** | | | | | | | |
| **Never** | 582/638 | 171/253 | 29.4/39.7 | 446/550 | 76.6/86.2 | **<0.001** | **0.79(0.71-0.87)** |
| **Ever** | 248/231 | 74/96 | 30.0/41.6 | 124/107 | 50.0/46.3 | **0.008** | **0.80(0.68-0.94)** |
| **Tea drinking** | | | | | | | |
| **Never** | 407/383 | 114/152 | 28.0/40.0 | 307/328 | 75.4/85.6 | **<0.001** | **0.76(0.67-0.86)** |
| **Ever** | 423/486 | 131/197 | 31.0/40.5 | 337/425 | 79.7/87.4 | **<0.001** | **0.81(0.72-0.91)** |
| **Family history of cancer** | | | | | | | |
| **No** | 639/747 | 182/297 | 28.5/39.8 | 495/645 | 77.5/86.3 | **<0.001** | **0.78(0.71-0.86)** |
| **Yes** | 191/122 | 63/52 | 33.0/42.6 | 149/108 | 78.0/88.5 | **0.037** | **0.80(0.65-0.99)** |
| **Hypertension** | | | | | | | |
| **No** | 614/667 | 188/267 | 30.6/40.0 | 477/575 | 77.7/86.2 | **<0.001** | **0.81(0.73-0.89)** |
| **Yes** | 216/202 | 57/82 | 26.4/40.6 | 167/178 | 77.3/88.1 | **<0.001** | **0.71(0.59-0.85)** |
| **Diabetes** | | | | | | | |
| **No** | 707/761 | 208/298 | 29.4/39.2 | 556/658 | 78.6/86.5 | **<0.001** | **0.81(0.73-0.88)** |
| **Yes** | 123/108 | 37/51 | 30.1/47.2 | 88/95 | 71.5/88.0 | **0.001** | **0.67(0.52-0.85)** |

Bold values indicated significant differences between two groups.

^a^Adjusted for age, bmi, pack-years of smoking, drinking status, tea drinking, hypertension and diabetes in logistic regression model.

**Supplementary Table 1D**. Stratification analysis of rs11874761 and PCa risk.

| rs11874761 | Case/Control | Genotypes | | | | P* | Adjusted OR(95%CI) ^a^ |
| --- | --- | --- | --- | --- | --- | --- | --- |
|  |  | GG | | AG+GG | |  |  |
| Variable |  | n | % | n | % |  | AG+GG vs. AA |
| **Total** | 830/869 | 649/676 | 78.2/77.8 | 818/858 | 98.6/98.7 | 0.836 | 0.99(0.88-1.11) |
| **Age** | | | | | | | |
| **≤71** | 383/429 | 303/336 | 79.1/78.3 | 379/423 | 99.0/98.6 | 0.712 | 1.03(0.87-1.22) |
| **>71** | 447/440 | 346/340 | 77.4/77.3 | 439/435 | 98.2/98.9 | 0.989 | 1.00(0.86-1.17) |
| **Smoke status** | | | | | | | |
| **Never** | 325/392 | 253/300 | 77.8/76.5 | 318/387 | 97.8/98.7 | 0.964 | 1.00(0.84-1.18) |
| **Ever** | 505/477 | 396/376 | 78.4/78.8 | 500/471 | 99.0/98.7 | 0.768 | 0.98(0.84-1.14) |
| **Pack-years of smoking** | | | | | | | |
| **0-20** | 229/232 | 184/182 | 80.3/78.4 | 229/230 | 100.0/99.1 | 0.730 | 1.04(0.83-1.31) |
| **>20** | 276/245 | 212/194 | 76.8/79.2 | 271/241 | 98.2/98.4 | 0.704 | 0.96(0.78-1.18) |
| **Drinking status** | | | | | | | |
| **Never** | 582/638 | 456/498 | 78.4/78.1 | 577/629 | 99.1/98.6 | 0.951 | 1.00(0.88-1.15) |
| **Ever** | 248/231 | 193/178 | 77.8/77.1 | 241/229 | 97.2/99.1 | 0.925 | 0.99(0.80-1.22) |
| **Tea drinking** | | | | | | | |
| **Never** | 407/383 | 323/297 | 79.4/77.5 | 404/378 | 99.3/98.7 | 0.565 | 1.05(0.89-1.24) |
| **Ever** | 423/486 | 326/379 | 77.1/78.0 | 414/480 | 97.9/98.8 | 0.514 | 0.95(0.81-1.11) |
| **Family history of cancer** | | | | | | | |
| **No** | 639/747 | 489/577 | 76.5/77.2 | 628/738 | 98.3/98.8 | 0.639 | 0.97(0.86-1.10) |
| **Yes** | 191/122 | 160/99 | 83.8/81.1 | 190/120 | 99.5/98.4 | 0.315 | 1.17(0.86-1.57) |
| **Hypertension** | | | | | | | |
| **No** | 614/667 | 488/516 | 79.5/77.4 | 604/657 | 98.4/98.5 | 0.625 | 1.03(0.91-1.18) |
| **Yes** | 216/202 | 161/160 | 25.5/20.8 | 214/201 | 99.0/99.5 | 0.242 | 0.87(0.69-1.10) |
| **Diabetes** | | | | | | | |
| **No** | 707/761 | 557/583 | 78.8/76.6 | 697/751 | 98.6/98.7 | 0.537 | 1.04(0.92-1.17) |
| **Yes** | 123/108 | 92/93 | 74.8/86.1 | 121/107 | 98.4/99.0 | 0.058 | 0.72(0.51-1.01) |

^a^Adjusted for age, bmi, pack-years of smoking, drinking status, tea drinking, hypertension and diabetes in logistic regression model.

**Supplementary Table 1E**. Stratification analysis of rs35996865 and PCa risk.

| rs35996865 | Case/Control | Genotypes | | | | P* | Adjusted OR(95%CI) ^a^ |
| --- | --- | --- | --- | --- | --- | --- | --- |
|  |  | TT | | GT+TT | |  |  |
| Variable |  | n | % | n | % |  | GT+TT vs. GG |
| **Total** | 830/869 | 635/699 | 76.5/80.4 | 814/854 | 98.1/98.2 | **0.040** | **0.89(0.80-0.99)** |
| **Age** | | | | | | | |
| **≤71** | 383/429 | 292/343 | 76.2/74.7 | 375/422 | 97.9/91.9 | 0.237 | 0.90(0.77-1.07) |
| **>71** | 447/440 | 343/356 | 76.7/80.9 | 439/432 | 98.2/98.2 | 0.166 | 0.90(0.77-1.05) |
| **Smoke status** | | | | | | | |
| **Never** | 325/392 | 249/311 | 76.6/79.3 | 317/383 | 97.5/97.7 | 0.223 | 0.90(0.76-1.07) |
| **Ever** | 505/477 | 386/388 | 70.5/81.3 | 497/471 | 98.4/98.7 | 0.095 | 0.88(0.75-1.02) |
| **Pack-years of smoking** | | | | | | | |
| **0-20** | 229/232 | 180/188 | 79.5/81.0 | 227/227 | 99.1/97.8 | 0.701 | 0.96(0.76-1.20) |
| **>20** | 276/245 | 206/200 | 74.6/81.6 | 270/244 | 97.8/99.6 | 0.107 | 0.84(0.68-1.04) |
| **Drinking status** | | | | | | | |
| **Never** | 582/638 | 448/516 | 77.0/80.9 | 575/628 | 98.8/98.4 | 0.128 | 0.90(0.79-1.03) |
| **Ever** | 248/231 | 187/183 | 75.4/79.2 | 239/226 | 96.4/97.8 | 0.311 | 0.90(0.73-1.10) |
| **Tea drinking** | | | | | | | |
| **Never** | 407/383 | 316/307 | 77.6/80.2 | 400/377 | 98.3/98.4 | 0.375 | 0.93(0.79-1.09) |
| **Ever** | 423/486 | 319/392 | 75.4/80.7 | 414/477 | 97.9/98.1 | 0.064 | 0.87(0.74-1.01) |
| **Family history of cancer** | | | | | | | |
| **No** | 639/747 | 481/604 | 75.3/80.9 | 626/735 | 98.0/98.4 | **0.011** | **0.85(0.75-0.96)** |
| **Yes** | 191/122 | 154/95 | 80.6/77.9 | 188/119 | 98.4/97.5 | 0.296 | 1.16(0.88-1.52) |
| **Hypertension** | | | | | | | |
| **No** | 614/667 | 474/533 | 77.2/80.0 | 602/655 | 98.0/98.2 | 0.204 | 0.92(0.81-1.05) |
| **Yes** | 216/202 | 161/166 | 74.5/82.2 | 212/199 | 98.1/98.5 | 0.074 | 0.81(0.64-1.02) |
| **Diabetes** | | | | | | | |
| **No** | 707/761 | 544/606 | 77.0/79.6 | 692/748 | 97.9/98.3 | 0.140 | 0.91(0.81-1.03) |
| **Yes** | 123/108 | 91/93 | 74.0/86.1 | 122/106 | 99.2/98.1 | **0.045** | **0.70(0.50-0.99)** |

Bold values indicated significant differences between two groups.

^a^Adjusted for age, bmi, pack-years of smoking, drinking status, tea drinking, hypertension and diabetes in logistic regression model.

**Supplementary Table 1F**. Stratification analysis of rs8089974 and PCa risk.

| rs8089974 | Case/Control | Genotypes | | | | P* | Adjusted OR(95%CI) ^a^ |
| --- | --- | --- | --- | --- | --- | --- | --- |
|  |  | TT | | GT+TT | |  |  |
| Variable |  | n | % | n | % |  | GT+TT vs. GG |
| **Total** | 830/869 | 648/702 | 78.1/80.8 | 822/855 | 99.0/98.4 | 0.204 | 0.93(0.83-1.04) |
| **Age** | | | | | | | |
| **≤71** | 383/429 | 298/351 | 77.8/81.8 | 379/421 | 99.0/98.1 | 0.412 | 0.93(0.78-1.11) |
| **>71** | 447/440 | 350/351 | 78.3/79.8 | 443/434 | 99.1/98.6 | 0.608 | 0.96(0.82-1.12) |
| **Smoke status** | | | | | | | |
| **Never** | 325/392 | 249/312 | 76.6/79.6 | 320/383 | 98.5/97.7 | 0.236 | 0.90(0.76-1.07) |
| **Ever** | 505/477 | 399/390 | 79.0/81.8 | 502/472 | 99.4/99.0 | 0.284 | 0.92(0.78-1.07) |
| **Pack-years of smoking** | | | | | | | |
| **0-20** | 229/232 | 184/188 | 80.3/81.0 | 229/232 | 100.0/100.0 | 0.798 | 0.97(0.76-1.23) |
| **>20** | 276/245 | 215/202 | 77.9/82.4 | 273/240 | 98.9/98.0 | 0.331 | 0.90(0.72-1.11) |
| **Drinking status** | | | | | | | |
| **Never** | 582/638 | 454/514 | 78.0/80.6 | 577/629 | 99.1/98.6 | 0.348 | 0.94(0.82-1.07) |
| **Ever** | 248/231 | 194/188 | 78.2/81.4 | 245/226 | 98.8/97.8 | 0.359 | 0.90(0.72-1.12) |
| **Tea drinking** | | | | | | | |
| **Never** | 407/383 | 314/304 | 77.1/79.4 | 404/376 | 99.3/98.2 | 0.608 | 0.96(0.81-1.13) |
| **Ever** | 423/486 | 334/398 | 79.0/81.9 | 418/479 | 98.8/98.6 | 0.256 | 0.91(0.78-1.07) |
| **Family history of cancer** | | | | | | | |
| **No** | 639/747 | 498/602 | 78.0/80.6 | 632/737 | 99.0/98.7 | 0.208 | 0.92(0.81-1.05) |
| **Yes** | 191/122 | 150/100 | 78.5/82.0 | 190/118 | 99.5/96.7 | 0.922 | 0.99(0.74-1.31) |
| **Hypertension** | | | | | | | |
| **No** | 614/667 | 485/541 | 79.0/81.1 | 608/655 | 99.0/98.2 | 0.376 | 0.94(0.82-1.08) |
| **Yes** | 216/202 | 163/161 | 75.5/79.7 | 214/200 | 99.0/99.0 | 0.376 | 0.90(0.71-1.14) |
| **Diabetes** | | | | | | | |
| **No** | 707/761 | 556/604 | 78.6/79.4 | 700/748 | 99.0/98.3 | 0.733 | 0.98(0.87-1.11) |
| **Yes** | 123/108 | 92/98 | 74.8/90.7 | 122/107 | 99.2/99.1 | **0.003** | **0.56(0.38-0.82)** |

Bold values indicated significant differences between two groups.

^a^Adjusted for age, bmi, pack-years of smoking, drinking status, tea drinking, hypertension and diabetes in logistic regression model.
